# Sex-Specific Weight Loss Mediates Sexual Size Dimorphism in *Drosophila melanogaster*

**DOI:** 10.1371/journal.pone.0058936

**Published:** 2013-03-28

**Authors:** Nicholas D. Testa, Shampa M. Ghosh, Alexander W. Shingleton

**Affiliations:** Department of Zoology and Ecology and Evolutionary Biology and Behavior, Michigan State University, East Lansing, Michigan, United States of America; MRC, University College of London, United Kingdom

## Abstract

The selective pressures leading to the evolution of Sexual Size Dimorphism (SSD) have been well studied in many organisms, yet, the underlying developmental mechanisms are poorly understood. By generating a complete growth profile by sex in *Drosophila melanogaster*, we describe the sex-specific pattern of growth responsible for SSD. Growth rate and critical size for pupariation significantly contributed to adult SSD, whereas duration of growth did not. Surprisingly, SSD at peak larval mass was twice that of the uneclosed adult SSD with weight loss between peak larval mass and pupariation playing an important role in generating the final SSD. Our finding that weight loss is an important regulator of SSD adds additional complexity to our understanding of how body size is regulated in different sexes. Collectively, these data allow for the elucidation of the molecular-genetic mechanisms that generate SSD, an important component of understanding how SSD evolves.

## Introduction

Sexual Size Dimorphism (SSD), the difference in body size between males and females, is an extraordinarily widespread and conspicuous phenomenon in the animal kingdom [1]. This condition is extremely variable and evolutionarily labile. For example, male southern elephant seals can weigh seven times that of a female [2], while female blanket octopi can weight 10,000-20,000 times their male counterparts [3]. The degree of SSD in insects is generally less extreme, however. Consistent with most invertebrates, the female is often the larger sex among insects, a pattern seen in approximately 88% of insect species [4]. Despite the ubiquity of SSD, however, very little is known of the underlying developmental mechanisms that generate it or how these mechanisms evolve.

In general, final body size is regulated by a combination of three developmental factors: initial body size (size at hatching/birth), growth rate, and growth duration [5], [6]. Changing any of these individually or in combination results in an alteration of adult body size and may underlie size differences between males and females. Nevertheless, the molecular-genetic and physiological regulators of initial size, growth rate, and growth duration are poorly understood except in a very few organisms. One such organism for which these mechanisms are known, is the fruit fly *Drosophila melanogaster,* which like most insects, shows marked SSD between female and male body size [4], [6]. The extensive research on growth regulation in *Drosophila* and its readily apparent SSD make these animals an ideal model to more deeply elucidate the proximate mechanisms that regulate SSD.

*Drosophila* are typical holometabolous insects: they begin life as worm-like larvae, molting through three larval instars before undergoing complete metamorphosis as a pupa and eventually eclosing into their adult form [7]. Adult flies, like all arthropods, have a stiff exoskeleton meaning they cannot grow. Larval body size upon termination of growth, therefore, ostensibly determines adult body size.

In *Drosophila,* the timing of metamorphosis is regulated by a larva reaching a size checkpoint called critical size (or critical weight) early in its final larval instar. Attainment of critical size is associated with initiation of a hormonal cascade that ends in metamorphosis. There is, however, temporal separation between the attainment of critical size and the subsequent rise in the ecdysteroid titer that causes the larva to stop feeding and ends body growth. This delay provides a final period of growth for the larvae, called the Terminal Growth Period (TGP), during which *Drosophila* larvae can more than triple their mass [8], [9]. Body size in *Drosophila* is therefore regulated by the critical size plus the amount of growth achieved during the TGP [8], [10]–[14], or more formally:
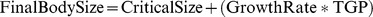
where Critical Size is the weight at which larvae commit to pupariation, TGP is the time between critical size and cessation of growth, and Growth Rate refers to the rate of growth within the TGP. SSD in *Drosophila* is therefore a consequence of sex-specific differences in one or all of these parameters.

Research over the last twenty years has begun to establish the developmental mechanisms that regulate critical size, growth rate and the duration of the TGP [15]–[18]. The goal of this study is to determine the proximate mechanisms responsible for SSD in *Drosophila melanogaster*. To test the hypothesis that sex-specific differences in a combination of developmental events underlies SSD, we measured critical size, growth rate, and growth duration for the developing larvae and pupae. Identifying how these developmental parameters differ between male and female flies therefore allows us to begin to link the observed SSD to the endocrine, and ultimately molecular-genetic, mechanisms that regulate growth and development.

## Materials and Methods

### 1. Fly Strains and Maintenance

All flies were derived from an isogenic stock of Samarkand (SAM) *Drosophila melanogaster*. Ubi-GFP (y^1^w^67c23^P{Ubi-GFP.D}ID-1) flies were obtained from Bloomington Stock Center and back crossed into a SAM background for five generations to eliminate background effects. Flies were raised on standard cornmeal-molasses food medium at 25°C on a 24 hour light cycle.

### 2. Critical Size

Mid-third instar larvae that weighed between 0.3 to 2.3 mg were placed into individual tubes and starved. Time to pupariation (TTP) was recorded on an individual basis and critical size was calculated as the weight at which starvation no longer delayed pupariation [see [9] for additional details]. Flies that survived to the late pupal period were sexed using the presence or absence of sex combs. For those pupae that died before the presence or absence of sex-combs could be scored, sex was determined by presence or absence of the Y-chromosome-specific Ppr-y gene using PCR and gel electrophoresis. DNA from un-sexable pupae extracted using a Qiagen DNA extraction kit and PCR was conducted under standard conditions. Optimal annealing temperature for PCR primers (below) was found to be 58°C.

Forward: 5′ TGT GTT GAT GAC CGT GAC GCC A 3′

Reverse: 5′ CGA GTC GCA ATT GTG TCT TCT CGC 3′

### 3. Growth rate

Eggs were laid in six-hour cohorts from which larvae were sampled every six hours and developmental stage and mass were recorded. Larval sex was determined by using presence or absence of a paternally inherited X-chromosome marked with a constituently active GFP. Sex was recorded based on presence or absence of GFP, to detect females and males respectively. Pupae were staged into four-hour cohorts at pupation and massed every 12 hours. Timing of pupariation was determined by using SAM flies laid in six-hour cohorts. Starting at 94 hours, we recorded pupariation state for individual larvae. Pupal sex was determined retrospectively by presence or absence of sex combs.

### 4. Statistical Analysis

All statistical analyses were conducted using R statistical software (version 2.14.1). Critical size was calculated using the methods described in Stieper et al. [9]. To assess the probability of observed sex-specific differences in critical size, we used a permutation test with one thousand replicates to generate a null distribution of the difference in critical size between males and females. The same test was also applied to determine differences in time to pupariation from the critical size data. Growth rate was calculated using a linear regression of log-transformed weight against time, while interactions with sex were tested using an Analysis of Covariance (ANCOVA). We calculated TGP by subtracting the time at which critical size is attained from the time at which larval weight no longer significantly increases, for each sex. We applied the values for critical size to the growth curve to determine the timing of critical size and used multiple comparisons analysis (Hsu's MCB) to determine the age at which there is no longer any significant increase in mass for each sex. Since this approach does not allow us to calculate 95% confidence intervals for the timing of growth cessation, confidence intervals for the duration of the TGP were predicted using those for critical size alone. In all larval cultures, we noticed that some larvae stopped growing prematurely and subsequently failed to pupariate. In order to avoid including these abnormal larvae in our growth calculations, any larvae whose weight was below critical size by the time the rest of the population had stopped growing were excluded from the analysis. Values for the timing of developmental stages were determined by logistic regression of developmental stage against age. Finally, all SSD indices were calculated as per the 1992 Lovich and Gibbons paper [19], [20], such that SSD  =  (F/M)-1, where F is female weight and M is male weight.

## Results

**Figure 1:**
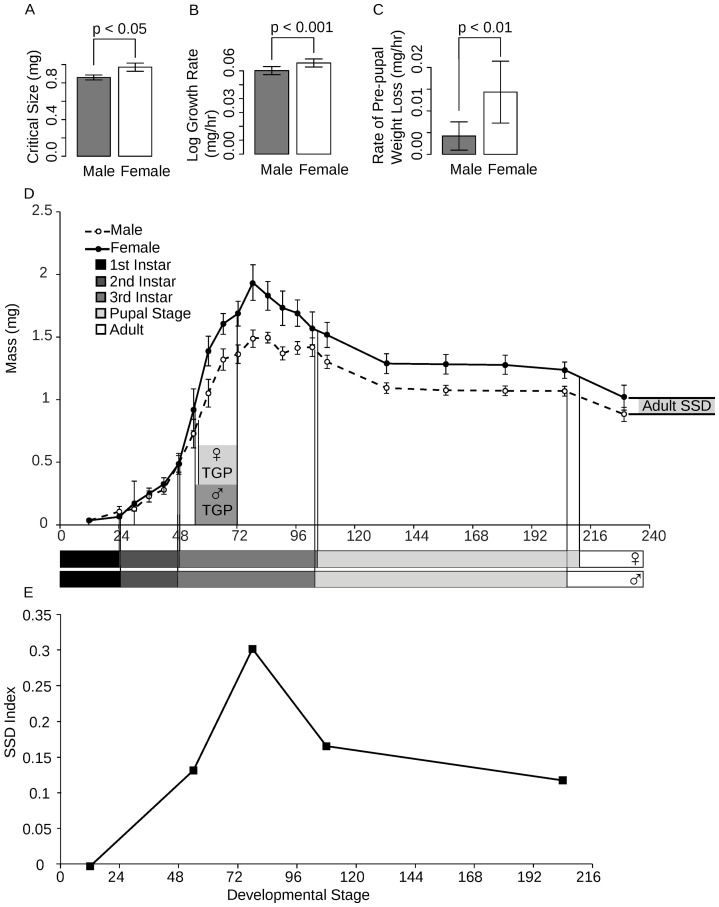
Complete growth profile by sex for *Drosophila melanogaster*. Factors shown to contribute to SSD include (a) critical size, (b) growth rate, (c) and pre-pupal weight loss and are reflected in the sex-specific growth curve (d). The SSD at specific developmental events (hatching, critical size, peak larval mass, pupariation and eclosion) illustrates the changes in SSD throughout development (e). Error bars show 95% confidence intervals.

There are three potential mechanisms by which SSD can be generated in *Drosophila*, namely, sex-specific differences in critical size, TGP, and growth rate. We found that male larvae have a significantly smaller critical size than females (permutation test, *P* = 0.008) (figure 1a). This does not, however, wholly explain the adult SSD. Females also grow more rapidly than males during their TGP (ANOVA, *P* = 0.0084) (figure 1b), although their TGPs are approximately the same duration (17.5 and 16 hours, respectively). The nature of our data does not allow us to test this statistically; however, males have a significantly longer time to pupariation from critical size than females (permutation test, *P* = 0.01), which is a proxy for the TGP. Finally, the timing of both larval and pupal molts as well as eclosion timing do not differ significantly between sexes (logistic regression; molt to 2nd instar, *P* = 0.5330*;* molt to 3rd instar, *P* = 0.8282; pupal molt, *P* = 0.7432; eclosion, *P* = 0.9628) (figure 1d).

Surprisingly, SSD at peak larval mass is twice that of the uneclosed adult fly: females were 30% larger than males at peak larval mass and 12% larger than males by end of pupal development (figure 1e). To determine why this difference in SSD exists, we measured pre-pupal weight loss, weight lost in the period intervening peak larval mass and pupariation, and pupal weight loss, weight lost during the pupal stage. The female rate of weight loss during the larval stage is significantly greater than the male rate (ANCOVA, *P* = 0.0116) (figure 1c), whereas there was no significant difference in pupal weight loss (ANCOVA, *P* = 0.6078).

## Discussion

Consistent with the females biased dimorphism in insects, female *Drosophila* adults are significantly larger than their male counterparts. Our data indicate that this sexual size dimorphism arises because females 1) initiate metamorphosis at a larger size than males, that is they have a larger critical size, and 2) grow faster than males in the terminal growth period between critical size and the cessation of larval growth. Surprisingly, however, the resulting SSD at the peak of larval mass is subsequently reduced before metamorphosis because females lose more mass during the pre-pupal period. Additionally, our data show that the timing of larval molts and pupation are nearly identical in males and females and that the duration of growth is not different between the sexes. Males do, however, eclose as adults slightly earlier than females.

There is a paucity of data concerning the patterns of growth that generate SSD in other insects [6], [21]–[23]. Perhaps, the best study has been in the tobacco hornworm, *Manduca sexta*, where critical size and the duration of the TGP (called the ICG in *M. sexta*) are important mechanisms contributing to SSD at the cessation of larval growth [21]. Additional studies indicate that SSD in other Lepidopterans accumulates during development primarily due to females adding more instars than males [24]. This is consistent with females having a longer TGP/ICG. In contrast, a previous study indicates that SSD among Drosophilidae is a consequence of sex-specific differences in growth rate and this is supported by our study [6]. Different insect species therefore appear to generate SSD using different developmental mechanisms. It is possible, however, that differences in SSD for both *Drosophila* and *Manduca* are a consequence of the differing environments in which each was reared.

The observation that SSD is influenced strongly by the loss of mass between the cessation of growth and pupation is a novel one, although post-eclosion weight loss has been implicated in regulating SSD in Lepidopterans [22], [23]. To a certain extent, mass loss after a larva has stopped feeding is an inevitable consequence of ongoing metabolic and developmental activity. What is not clear is why females lose more mass than males; it seems counterintuitive for females to accrue mass only to lose it. One hypothesis is that selection for larger female size targets a systemic increase in growth rate, both of the body as a whole, but also of the imaginal discs, the precursors of adult organs. Importantly, growth and development of the imaginal discs continues after the cessation of feeding [25], [26], relying on stored nutrients to proceed [27], [28]. Thus, we might expect that larger females with larger organs will utilize more of these stored nutrients during post-feeding imaginal disc growth. Consequently, both the increase in the body's growth rate before cessation of feeding and the increase in weight loss after the cessation reflect the same mechanisms of elevated growth rate for increased body size in females.

**Figure 2:**
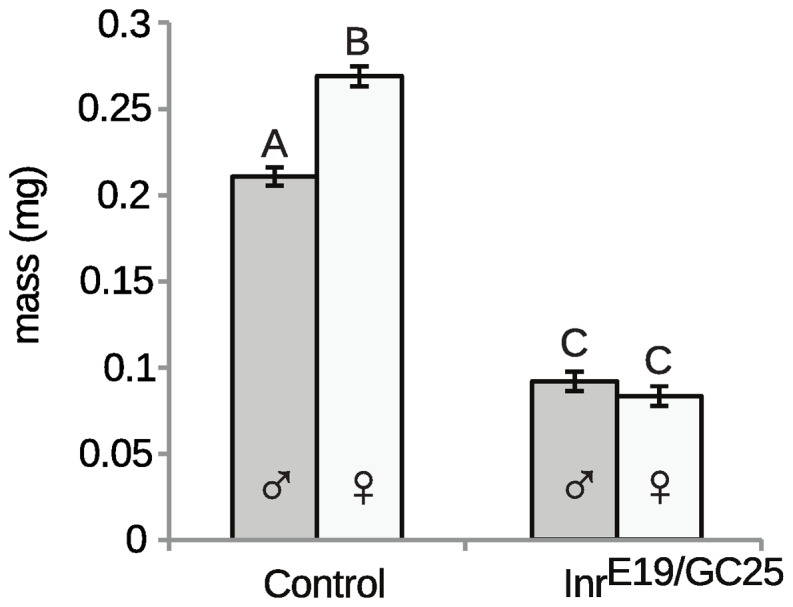
SSD is lost in insulin-signaling mutants. The dry mass of male and female adult Inr^E19^/Inr^GC25^ and wild-type (Inr^E19^/TM3) control flies reared at low density at 24°C. Columns with different letters are significantly different (Tukey HSD at P<0.05). Error bars are standard errors.

There are a number of pathways that control growth rate, which include IIS, TOR, MAPK, and HIF-signaling pathways [29]–[33]. Of these, the insulin signaling pathway has been demonstrated to have an important role in regulating final body size [34]–[36]. This pathway regulates the rate of cell growth and proliferation in response to insulin-like peptides that are released in a nutrient dependent manner by the brain and other tissue around the body [33], [37], [38]. Ostensibly, therefore, insulin signaling regulates growth and final body size with respect to developmental nutrition. Data from *Drosophila* and other animals, however, suggest that differences in insulin signaling may account for body size variation among different populations [39]–[41], suggesting that it may be a more general regulator of size. An intriguing hypothesis therefore, is that female *Drosophila* are larger than males because elevated levels of insulin signaling increases growth rate. The insulin signaling hypothesis was first proposed to explain SSD in *Manduca sexta* [4], however, evidence suggests that it may be important in regulating SSD in *Drosophila* as well. Support for this hypothesis comes from the observation that SSD is eliminated in flies mutant for the insulin receptor (*Inr*) (figure 2), indicating that insulin signaling is necessary to generate size differences between males and females. The fact that there is no SSD in *Inr* mutants, however, suggests that insulin signaling is also regulating other mechanisms that generate SSD, specifically difference in critical size.

Regardless of the function of pre-pupal weight loss, our understanding of how body size is regulated in *Drosophila melanogaster* needs to be extended. Pre-pupal weight loss should now be viewed as an additional variable for calculating final body size, such that:
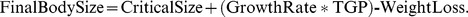



In conclusion, our data suggest that the mechanisms regulating critical size and growth rate are responsible for generating SSD in *Drosophila melanogaster*. Our understanding of the underlying molecular-genetic mechanisms that regulate these processes indicate that these studies can be extended to generate a deeper understanding of the development of SSD.
